# Impact on daily mobility and risk of falling in bilateral vestibulopathy

**DOI:** 10.1007/s00415-022-11043-9

**Published:** 2022-03-14

**Authors:** M. Wuehr, J. Decker, F. Schenkel, K. Jahn, R. Schniepp

**Affiliations:** 1 German Center for Vertigo and Balance Disorders, University of Munich, Munich, Germany; 2 Schön Klinik Bad Aibling, Bad Aibling, Germany; 3 Department of Neurology, University of Munich, Munich, Germany

**Keywords:** Bilateral vestibulopathy, Daily mobility, Falls, Body worn senor, Wearable

## Abstract

**Objective:**

To study the behavioral relevance of postural and ocular-motor deficits on daily activity and risk of falling in patients with bilateral vestibular hypofunction (BVH).

**Methods:**

Thirty patients with BVH and 30 age- and gender-matched healthy controls participated in a continuous 2-week assessment of daily activities and mobility using a body-worn inertial sensor and a 6-month prospective fall risk assessment. At inclusion, patients and controls further underwent a multi-modal clinical, score- and instrument-based assessment of general health and balance status. We analyzed the relationship between clinical, lab-, and sensor-based measures and their validity to identify those patients at a risk of general, frequent, and severe falling.

**Results:**

Patients exhibited impairments in daily activity in particular in terms of reduced ambulatory activity (*p* = 0.009). 43% of patients experienced falls (13% in controls, *p* = 0.008) and 70% of these patients reported recurrent falling (0% in controls, *p* = 0.001) during prospective assessment. Severe fall-related injuries that would require medical attention neither occurred in patients nor in controls. Classificatory models based on multi-modal clinical, lab-, and sensor-based measures of balance and mobility identified patients who fell with an accuracy of 93% and patients who recurrently fell with an accuracy of 89%.

**Conclusion:**

BVH is linked to particular impairments of patients’ daily activities which in turn are related to patients’ fall risk. Hence, off-laboratory measures of daily mobility may supplement standard clinical assessment in BVH to more adequately capture the burden of disease and to reliably identify those patients at a specific risk of falling.

## Introduction

The peripheral vestibular system senses motion and orientation of the head with respect to gravity and provides a central reference frame for spatial orientation, regulation of upright posture, and stabilization of gaze during head movements [1]. The primary complaints of patients with a chronic bilateral vestibular hypofunction (BVH) are consequently related to a chronic postural unsteadiness during stance and gait [2], an impaired gaze stabilization with oscillopsia during head turns and locomotion [3], and deficits in spatial memory and navigation [4]. Deficits in the balance and ocular-motor domain in BVH have been further associated to an increased risk of falling [5–7] and a reduced quality of life [8].

Postural and ocular-motor deficits in patients with BVH were hitherto primarily characterized within clinical settings [9–11]. Clinical and lab-based measures are consequently currently the primary endpoints of choice for clinical trials in patients with BVH [12–15]. However, it is not well understood how these measures relate to limitations in patients’ daily-life activities and whether they sufficiently capture the actual disease burden in BVH. Furthermore, a lab-based assessment of stance and gait deficits may potentially underestimate the challenges of daily-life mobility under which falls actually occur and might therefore yield a biased evaluation of patients’ individual risk of falling.

As of recently, advances in real-world mobility assessment based on body-worn inertial sensors offer the possibility to directly study the impact of BVH-related impairments on daily-life activities and mobility in individual patients [16]. Whereas lab-based measures commonly focus on the detailed spatiotemporal features of balance and gait deficits, measures from body-worn sensors provide complementary insights into a patient’s overall mobility status and balance capabilities from a macroscopic perspective [17, 18]. This information could entail important information to elucidate the ecological relevance of vestibular hypofunction for patients’ activities of daily living and mobility, to establish new clinical endpoints for future clinical trials in BVH, and to supplement currently available screening tools for fall risk assessment in BVH.

The aim of this study was to examine the impact of BVH-related impairments on patients’ daily mobility and risk of falling. To this end, we prospectively assessed patterns of daily activities and mobility as well as the circumstances, consequences, and frequency of falling in patients with BVH and healthy controls. Based on this assessment, we analyzed the relationship between multi-modal in- and off-laboratory measures of health and mobility status in patients and evaluated their relevance for forecasting the risk of falling on an individual patient level.

## Methods

### Participants

Thirty patients with BVH and 30 healthy controls (Table 1) were recruited as part of a large-scale prospective study (study ID: DRKS00007762). All patients showed a clinically proven deficit, i.e., a bilateral pathological head impulse test (horizontal gain < 0.6) and/or bilateral reduced or absent caloric responses (sum of maximal peak velocities of the slow-phase nystagmus with cold and warm water < 0.6°/s) [19]. Further inclusion criteria were the ability to ambulate independently and the absence of any manifest motor weakness of the lower limbs (hemiparesis, paraparesis of the legs). Relatives of patients and employees at the hospital were recruited as healthy controls. Participants gave their informed written consent prior to inclusion.

**Table 1:** Demographics, clinical characteristics, and falls epidemiology

	Patients with BVH (*N* = 30)	Healthy controls (*N* = 30)
Age (years)	55.9 ± 19.4 range: [20; 87]	51.2 ± 15.6 range: [28; 79]
Gender (female | male)	15 | 15	15 | 15
Etiology	11 idiopathic, 2 Ménière’s disease, 4 antibiotics, 13 other causes	
Neuro-otological characteristics		
vHIT gain (left–right average)	0.32 ± 0.19	–
vHIT gain asymmetry (%)	25.0 ± 21.1	–
Caloric response (°/s; left–right average)	5.7 ± 7.2	–
Presence of peripheral neuropathy (yes | no)	4 | 26	0 | 30
Retrospective fall status (N)		
Faller (yes | no)	10 | 20	3 | 27
Frequent faller (yes | no)	5 | 25	0 | 30
HFGS (1 | 2 | 3 | 4)	12 | 5 | 2 | 3	5 | 1 | 2 | 0
Severe faller (yes | no)	5 | 25	2 | 28
Prospective fall status (N)		
Number of falls | near-falls	36 | 176	4 | 23
Faller (yes | no)	13 | 14	4 | 23
Frequent faller (yes | no)	9 | 18	0 | 27
HFGS (1 | 2 | 3 | 4)	5 | 13 | 0 | 0	2 | 4 | 0 | 0
Severe faller (yes | no)	0 | 27	0 | 27

*vHIT* video head impulse test; *HFGS* Hopkins Falls Grading Scale

### Clinical assessment

The degree of vestibulopathy in each patient was documented using video-oculography (EyeSeeTec GmbH, Munich, Germany) with respect to the gain and side asymmetry of eye responses during video head impulse test (vHIT) as well as the left–right average of the sum of the maximal slow-phase eye movements during cold and warm water caloric irrigation test [19]. All participants were further clinically screened for signs of peripheral neuropathy (i.e., reduced vibrotactile thresholds and ankle jerk reflexes).

All participants took part in a standardized interview, which included an inquiry of the following information: ambulatory status, functional status, medication. As part of a retrospective fall risk assessment, information on the number and the severity of falls within the past 6 months (based on the Hopkins Falls Grading Scale (HFGS): no falling = grade 0; near falling = grade 1; falling without requiring medical attention = grade 2; falling requiring medical attention = grade 3; falling requiring admission to the hospital = grade 4; [20]) was collected.

The subjective level of balance confidence was evaluated by the Falls Efficacy Scale-International (FES-I) and the Activities-specific Balance Confidence scale (ABC-d) [21, 22]. Health-related quality of life was assessed by the Short-Form Health Survey (SF-12) [23]. Cognitive function was screened with the Montreal Cognitive Assessment (MoCA) [24]. Each participant underwent a complete neurological and physical examination including the assessment of functional mobility by the Timed up and Go test (TUG) and the Functional Gait Assessment score (FGA) [25, 26].

### Prospective fall assessment

Each participant was provided with a falls diary covering a 6-month follow-up period. Participants were asked to document near-fall and fall events on a daily basis with information on the time, the environmental circumstances, the fall mechanism (e.g., tripping, vertigo/dizziness, impaired consciousness, and others), the duration of the post-fall lying phase, and the related HFGS of each event. Participants were additionally contacted by phone on a monthly basis to cross-check and validate the documented information. Based on the prospective fall assessment, participants were categorized with respect to fall status (non-faller vs. faller), frequency (non-frequent vs. frequent faller, i.e., two or more falls during follow-up), and severity (falls that do, i.e., HFGS 3 or 4, vs. do not necessitate medical attention, i.e., HFGS 1 or 2).

### In-laboratory gait examination

In-laboratory gait assessment was performed on a 6.7-m-long pressure-sensitive carpet (GAITRite®, CIR System, Sparta, NJ, USA) at 120 Hz. Participants walked over the carpet at their preferred (PWS), slow (SWS), and maximum walking speed (MWS). Each condition was recorded 4 times. Gait assessment was conducted without additional ambulatory aids. For each walking condition, 13 spatiotemporal gait parameters representative for five established domains of walking [17] were analyzed: (1) *pace* domain: gait velocity, stride length, stride time; (2) *phase* domain: swing and double support phases; (3) *variability* domain: coefficient of variation (CV) of stride length, stride time, swing phase; (4) *asymmetry* domain: left–right asymmetry of stride length, stride time, swing phase, (5) *postural control* domain: mean and CV of base of support.

### Daily mobility assessment

Monitoring of daily mobility was undertaken for 14 days. Participants wore an inertial-sensor-based activity monitor (ActivPAL®, PAL Technologies, Glasgow), which recorded the sequence and period of time of individual bouts of ambulatory and sedentary behavior at a sampling rate of 10 Hz. The inertial sensor was placed at the thigh of the dominant leg approximately 0.1 m cranial and 0.05 m lateral of the patella. Participants were advised to continue their daily activities as usual and not to change their routine. At the end of the recording period, participants removed the sensor independently and sent it back via postal service.

The following standard parameters (expressed as average daily estimates of ambulatory and sedentary behavior) were computed from the ActivPAL recordings to represent five previously established domains of daily activity and mobility [18, 27]: (1) *ambulatory volume* domain: intensity (i.e., the amount of energy expenditure expressed as the total metabolic equivalents, METS), percentage of ambulatory time, number of ambulatory bouts and total step count; (2) *ambulatory pattern* domain: the average duration, variability, and distribution of ambulatory bouts; (3) *postural transitions* domain: the number of sit-to-stance transitions, the number and average duration of sedentary bouts; (4) *sedentary volume* domain: the percentage of sedentary time; (5) *sedentary pattern* domain: the variability and distribution of sedentary bouts. The distribution of ambulatory/sedentary bouts was computed as the exponent alpha, with lower alpha values indicating a greater contribution of long bouts [28].

### Statistical analysis

Descriptive statistics are reported as mean ± standard deviation (SD). Group differences between patients and controls in clinical, gait, and daily mobility characteristics were analyzed by a one-way analysis of variance (ANOVA). Group differences in fall characteristic (status, frequency, severity) were analyzed by Pearson’s Chi-squared test. Determinants of patients’ daily mobility measures (clinical and gait characteristics) were analyzed by Pearson’s correlation coefficient. Finally, linear discriminant analysis with cross-validation was used to build classificatory models for patient’s fall status based on clinical, gait, and daily mobility characteristics. Results were considered significant if *p* < 0.050. Statistical analysis was performed using SPSS (version 26.0, IBM corp., Armonk, NY).

## Results

### Clinical characteristics

**Fig. 1:**
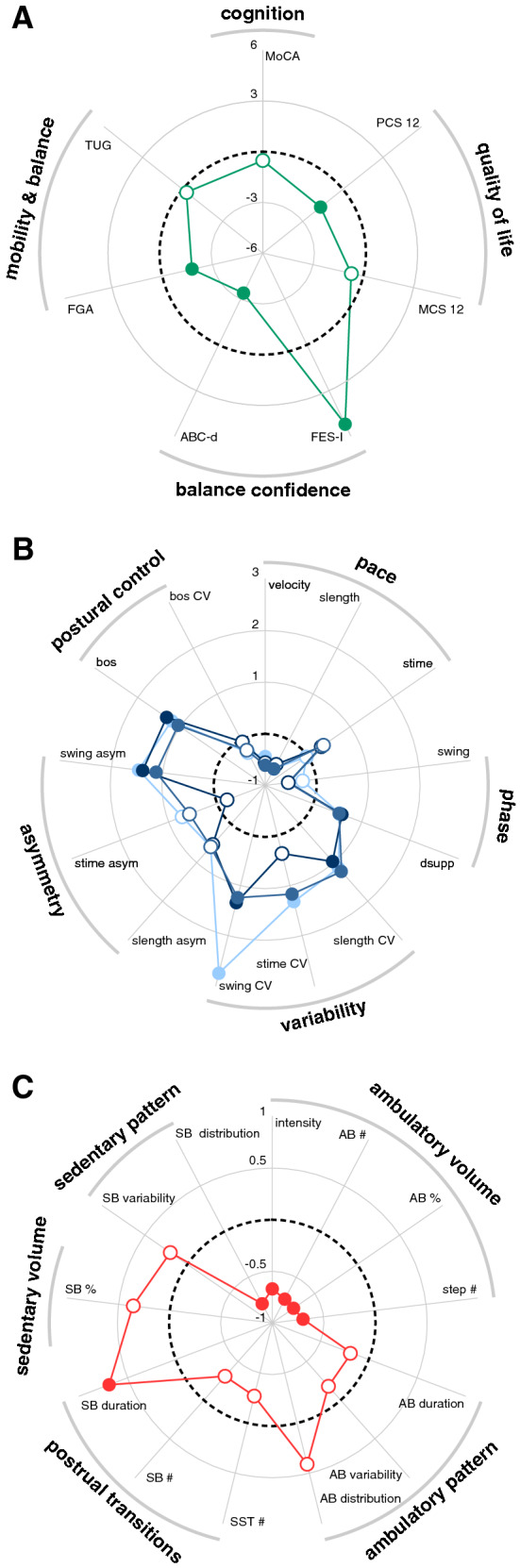
Impact of bilateral vestibulopathy on clinical, gait, and daily mobility measures. Radar plots with mean *z*-values of patients’ **A** clinical (*green*), **B** in-laboratory gait (slow speed: *light blue*; preferred speed: *medium blue*; fast speed: *dark blue*), and **C** off-laboratory mobility characteristics (red) normalized with respect to healthy controls (*dotted black line*). *Filled circles* indicate a significant difference compared to healthy controls. MoCA, Montreal Cognitive Assessment; PCS 12 & MCS 12, physical and mental component score of the Short-Form Health Survey; FES-I, Falls Efficacy Scale-International; ABC-d, Activities-specific Balance Confidence scale; TUG, Timed up and Go test; FGA, Functional Gait Assessment; slength, stride length; stime, stride time; swing, swing percentage; dsupp, double support percentage; bos, base of support; CV, coefficient of variation; asym, asymmetry; AB, ambulatory bout; SB, sedentary bout; SST, sit-to-stance transition

Demographic information and clinical characteristics of patients and controls are presented in Table 1. Clinical assessment (Fig. 1A) of functional mobility revealed impaired balance and gait performance in the FGA (*F*_1,59_ = 24.3; *p* < 0.001) but not in the TUG test. Correspondingly, patients reported increased fear of falling (FES-I: *F*_1,59_ = 34.7; *p* < 0.001), a lower balance confidence (ABC-d: *F*_1,59_ = 47.7; *p* < 0.001), and reduced quality of life scores with respect to physical function (physical component scale of the SF-12, *F*_1,59_ = 25.4; *p* < 0.001) but showed normal quality of life scores with respect to mental function (mental component scale of the SF-12) and age-appropriate cognitive function (MoCA).

### In- and off-laboratory gait and mobility assessment

In-laboratory gait assessment (Fig. 1B) revealed gait alterations in patients with BVH compatible with a sensory-ataxic gait disorder. Accordingly during preferred walking, patients walked with reduced speed (*F*_1,59_ = 6.8; *p* = 0.012) and stride length (*F*_1,59_ = 6.0; *p* = 0.017), reduced swing (*F*_1,59_ = 5.1; *p* = 0.027) and increased double support (*F*_1,59_ = 4.6; *p* = 0.036) phases, increased spatiotemporal gait variability (stride length CV: *F*_1,59_ = 11.7; *p* = 0.001; stride time CV: *F*_1,59_ = 10.5; *p* = 0.002; swing phase CV: *F*_1,59_ = 14.5; *p* < 0.001) and gait asymmetry (swing phase asymmetry: *F*_1,59_ = 13.9; *p* < 0.001), and increased base of support (*F*_1,59_ = 16.0; *p* < 0.001). These walking abnormalities became even more pronounced during slow walking but tended to normalize during fast walking.

Off-laboratory assessment of daily activity and mobility (Fig. 1C) revealed a general reduction in the *ambulatory volume* domain, in terms of a reduced overall daily intensity of activity (*F*_1,59_ = 7.1; *p* = 0.010), percentage of ambulatory activity (*F*_1,59_ = 7.4; *p* = 0.009), number of ambulatory bouts (*F*_1,59_ = 4.3; *p* = 0.042), and number of steps (*F*_1,59_ = 7.3; *p* = 0.009). Patients further exhibited altered mobility behavior in the domains *postural transition* (increased average sedentary bout duration: *F*_1,59_ = 5.6; *p* = 0.021) and *sedentary pattern* (increased amount of long sedentary bouts: *F*_1,59_ = 6.6; *p* = 0.013). Near-to-normal performance was found for the mobility domains *ambulatory pattern* and *sedentary volume*. Alterations in patients’ daily activity and mobility were neither associated with age nor clinical outcomes of vestibular function and balance examination nor measures from in-laboratory gait assessment.

### Fall assessment

Information on fall epidemiology from retro- and prospective fall risk assessment is summarized in Table 1. Both retro- and prospective assessment revealed a significantly higher incidence of fallers and recurrent fallers in the cohort of patients with BVH compared to healthy controls. With respect to retrospective assessment, 33% of patients had experienced at least one fall within the last 6 months (10% in controls, χ_1,60_ = 4.8, *p* = 0.028), 50% of those reported recurrent falling (0% in controls, χ_1,60_ = 5.5, *p* = 0.020), and also 50% of those reported severe fall-related injuries (HFGS 3 or 4; 67% in controls).

In the 6-month follow-up period, all participants registered falls information in their falls diary that was counter-checked by structured monthly telephone interviews. Falls diary information from 3 patients and 3 controls was considered invalid and excluded from further analysis due to missing telephone contact data or discrepancies between the information documented in the diary and the information surveyed during monthly interviews. The total number of near-fall and fall events in patients registered during prospective assessment were 176 and 36, respectively. Falls equally occurred indoors or outdoors with no difference for domestic or non-domestic environments. Patients fell primarily on even surface (61%). Most falls were linked to walking or turning (60%) and 65% occurred either forwards or backwards in anterior–posterior direction. The most frequent causes of falling were imbalance (31%) and tripping (22%). The most commonly reported symptoms associated with falling were dizziness and vertigo (48%). Overall, outcomes from prospective assessment revealed that 43% of the patients experienced falls within the 6-month follow-up period (13% in controls, χ_1,54_ = 7.0, *p* = 0.008) and 70% of these patients reported recurrent falling (0% in controls, χ_1,54_ = 10.8, *p* = 0.001). Severe fall-related injuries that required medical attention neither occurred in patients nor healthy controls during the 6-month follow-up period.

### Fall status classification in patients

**Fig. 2:**
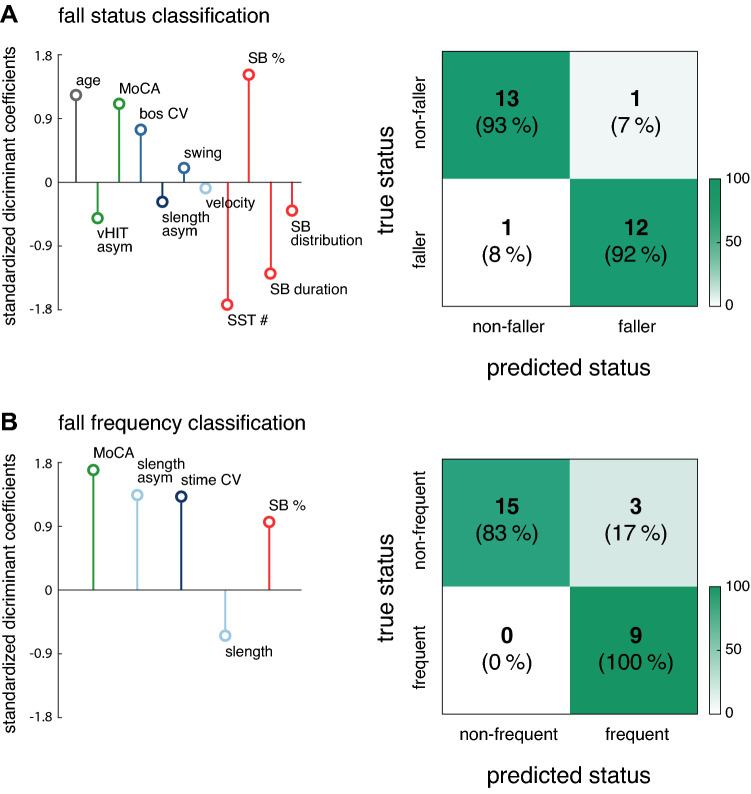
Classification of fall status and frequency in patients with bilateral vestibulopathy. Classification models for patients’ fall status **A** and fall frequency **B**. Left panel: standardized canonical discriminant coefficients from demographic (*grey*), clinical (*green*), gait (slow walking: *light blue*; preferred walking: *medium blue*; fast walking: *dark blue*), and daily mobility (*red*) characteristics. *Right panel*: corresponding confusion matrices. MoCA, Montreal Cognitive Assessment; slength, stride length; stime, stride time; swing, swing percentage; bos, base of support; CV, coefficient of variation; asym, asymmetry; SB, sedentary bout; SST, sit-to-stance transition

Using linear discriminant analysis, classificatory models based on demographic, clinical, gait, and daily mobility characteristics were built for patients' prospectively assessed fall status and fall frequency. No model was built for fall severity since none of the patients fell severely during follow-up. The model for fall status identified patients who fell during follow-up with a cross-validated accuracy of 93% (sensitivity: 92%; specificity: 93%; Wilks Λ = 0.001, *p* < 0.001) based on age and 10 clinical, gait, and daily mobility characteristics (Fig. 2A). The model for fall frequency identified patients who fell frequently (i.e., two or more falls during follow-up) with a cross-validated accuracy of 89% (sensitivity: 100%; specificity: 83%; Wilks Λ = 0.113, *p* < 0.001) based on 5 clinical, gait, and daily mobility characteristics (Fig. 2B).

## Discussion

In this study, we examined patterns of daily mobility impairments and falling in patients with BVH. We found that postural, ocular-motor, and orientational deficits associated to chronic vestibular hypofunction have relevant consequences for patients’ everyday behavior that can be objectified by means of a sensor-based activity monitoring. Long-term mobility measures accordingly revealed that patients with BVH are prone to significantly reduce their daily activities and become more sedentary. By means of a prospective fall assessment, we further provide a detailed characterization of the circumstances and mechanisms of falling in these patients and demonstrate that they are at a particular risk for recurrent but not for injurious falling. We finally explored the determinants for falling in BVH and established predictive models that allow to reliably identify patients at risk of falling based on clinical information and measures from in- and off-laboratory gait and mobility assessment. In the following, we will discuss these findings with respect to mobility impairments, circumstances and mechanisms of falling as well was fall risk prediction in patients with BVH.

### Impact of bilateral vestibulopathy on daily mobility

During clinical evaluation, patients from our study cohort presented with moderate balance impairments and a staggering, broad-based walking pattern consistent with a vestibular ataxic balance and gait disorder [2, 7]. Previous interview-based surveys in patients with BVH already indicated a general negative impact of imbalance and dizziness on patients' daily activities, including more frequent absence from work and a reduced productivity [29]. Our long-term sensor-based activity monitoring complements these earlier reports and allows to objectify the impact of chronic vestibular hypofunction on daily activities. Accordingly, we found that patients with BVH exhibit moderate reductions (− 20–30% compared to the age-matched healthy population) in their total volume and intensity of ambulatory activities linked to increasingly long periods of sedentary behavior over the day. This observation suggests that patients with BVH in general retain the ability to independently manage their daily duties but are prone to adopt a progressively sedentary lifestyle.

This negative impact of vestibulopathy on physical activity is reflected in the observed lower quality of life scorings of patients in particular with respect to physical functioning [8]. Above that, an increased sedentary behavior has been associated to a variety of poor health outcomes (obesity, diabetes, hypertension, etc. [30]) and may thus present an independent risk factor for severe secondary comorbidities in patients with BVH. It is natural to suppose that the observed limitations of daily-life activity in patients with BVH are determined by both their postural unsteadiness and gait impairment as well as their resultant lowered balance confidence and increased fear of falling. However, we did not find convincing evidence for this linkage in our cohort, presumably due to the rather small size of the studied sample of patients.

### Circumstances and mechanisms of falling in bilateral vestibulopathy

The outcomes from our 6-month prospective fall assessment largely support previous retrospective surveys on fall risk in BVH [6, 7, 31]. Patients with BVH correspondingly suffer from a general higher incidence of falling and experience considerably more frequently recurrent falling compared to the age-matched healthy population. Fall incidences from prospective assessment even exceed those from retrospective interrogation which is likely caused by the well-known recall bias often observed in retrospective surveys [32].

Patients with BVH consistently report that their postural imbalance and dizziness worsen in darkness and/or on uneven ground, i.e., in circumstances where sensory feedback from the remaining intact senses becomes unreliable. In contrast, we found that falls in BVH are not linked to specific environments and/or circumstances but occur on all kinds of support surfaces and are equally likely to happen in outdoor or domestic and non-domestic indoor environments. Rather than a dependence on specific circumstances, we found that most falls were associated to locomotor activities such as walking or turning and were caused by either imbalance or tripping. Similar to falls in cerebellar ataxic gait disorders [33], this suggests that most falls in patients with BVH are intrinsically generated in situations where the center of mass rapidly moves outside the base of support and dynamic balance cannot be timely and sufficiently recovered. In contrast to patients with cerebellar ataxia, however, patients with BVH are apparently at a low risk to experience injurious falls, which indicates that they retain sufficient postural strategies to reduce and/or neutralize the impact forces when falling.

### Fall risk prediction in bilateral vestibulopathy

To identify explanatory variables that may predict the general fall status and fall frequency in patients with BVH, we performed a linear discriminant analysis considering a broad range variables from sociodemographic characteristics, clinical vestibular function tests and scores as well as quantitative measures from in- and off-laboratory gait and mobility assessment. The resultant classificatory models yielded an excellent cross-validated accuracy of about 90% that outperforms previous approaches which only considered a limited set of explanatory characteristics [7, 31].

We found, that in particular macroscopic mobility measures from daily activity monitoring have the strongest impact on predicting the general fall status of patients. Accordingly, patients who extensively adopt a sedentary lifestyle and perform only few postural transitions between seated and upright postures over the day are specifically prone to fall. Less time spent during ambulatory activities in these patients might involve a reduced practice to perform adequate postural maneuvers that restabilize posture in the case of a trip or a sudden instability during walking. On the other side, measures from in-laboratory gait assessment become increasingly relevant for identifying patients at the risk of recurrent falling. Hence, the extent of ataxic fluctuations and asymmetries in their walking pattern appears to mainly determine whether patients will repeatedly fall and thus be at a permanent risk of serious injury. In addition, we found an expectable link between advanced age and the general fall risk. Noteworthy, outcomes from clinical vestibular function testing showed only a weak association to fall risk. Finally, we found that the cognitive status of patients was predictive for the general fall risk and the risk of recurrent falling. A decline in cognitive recourses is commonly considered an independent risk factor for falling [34]. In contrast, our classificatory models suggest an inverse relationship in that patients with better cognitive performance were at a higher general risk of falling and of experiencing recurrent falling. This finding suggests that patients with moderate cognitive impairments might expose themselves less frequently to complex balance situations and thus be more protected against falling compared to patients with intact cognitive recourses [35, 36].

## Conclusions

Our current findings emphasize the impact of chronic vestibular hypofunction on daily activities and mobility in afflicted patients. Real-world activity monitoring in these patients entails important complementary information on patients’ mobility status and fall risk that is not readily available form clinical evaluation. Quantitative measures on patients’ daily mobility might thus be ecological relevant and suitable endpoints for future clinical trials on developing therapeutic approaches in BVH [14, 37, 38]. Finally, we observed that falls in BVH are tightly linked to an impaired locomotor function and can be reliably predicted when considering quantitative measures from in- and off-laboratory gait and mobility assessment in these patients.
